# Ruminant-specific multiple duplication events of PRDM9 before speciation

**DOI:** 10.1186/s12862-017-0892-4

**Published:** 2017-03-14

**Authors:** Abinash Padhi, Botong Shen, Jicai Jiang, Yang Zhou, George E. Liu, Li Ma

**Affiliations:** 1 Department of Animal and Avian Sciences, University of Maryland, College Park, MD 20742 USA; 2 Animal Genomics and Improvement Laboratory, Agricultural Research Service, USDA, Beltsville, MD 20705 USA; 3 College of Animal Science and Technology, Northwest A & F University, Shaanxi Key Laboratory of Agricultural Molecular Biology, Yangling, Shaanxi 712100 China

**Keywords:** Speciation genetics, Genetic incompatibility, PRDM9, Gene duplication, Positive selection, Molecular evolution, Ruminants

## Abstract

**Background:**

Understanding the genetic and evolutionary mechanisms of speciation genes in sexually reproducing organisms would provide important insights into mammalian reproduction and fitness. PRDM9, a widely known speciation gene, has recently gained attention for its important role in meiotic recombination and hybrid incompatibility. Despite the fact that PRDM9 is a key regulator of recombination and plays a dominant role in hybrid incompatibility, little is known about the underlying genetic and evolutionary mechanisms that generated multiple copies of PRDM9 in many metazoan lineages.

**Results:**

The present study reports (1) evidence of ruminant-specific multiple gene duplication events, which likely have had occurred after the ancestral ruminant population diverged from its most recent common ancestor and before the ruminant speciation events, (2) presence of three copies of PRDM9, one copy (lineages I) in chromosome 1 (chr1) and two copies (lineages II & III) in chromosome X (chrX), thus indicating the possibility of ancient inter- and intra-chromosomal unequal crossing over and gene conversion events, (3) while lineages I and II are characterized by the presence of variable tandemly repeated C2H2 zinc finger (ZF) arrays, lineage III lost these arrays, and (4) C2H2 ZFs of lineages I and II, particularly the amino acid residues located at positions −1, 3, and 6 have evolved under strong positive selection.

**Conclusions:**

Our results demonstrated two gene duplication events of PRDM9 in ruminants: an inter-chromosomal duplication that occurred between chr1 and chrX, and an intra-chromosomal X-linked duplication, which resulted in two additional copies of PRDM9 in ruminants. The observation of such duplication between chrX and chr1 is rare and may possibly have happened due to unequal crossing-over millions of years ago when sex chromosomes were independently derived from a pair of ancestral autosomes. Two copies (lineages I & II) are characterized by the presence of variable sized tandem-repeated C2H2 ZFs and evolved under strong positive selection and concerted evolution, supporting the notion of well-established Red Queen hypothesis. Collectively, gene duplication, concerted evolution, and positive selection are the likely driving forces for the expansion of ruminant PRDM9 sub-family.

**Electronic supplementary material:**

The online version of this article (doi:10.1186/s12862-017-0892-4) contains supplementary material, which is available to authorized users.

## Background

Ever since the theory of genetic incompatibility (Bateson-Dobzhansky-Muller Model) was independently proposed by three eminent evolutionary biologists [1–3], researchers across the disciplines have been devoted to characterizing the evolutionary impacts of reproduction-associated genes on speciation and species diversity. Understanding the molecular diversity of speciation genes would unravel the underlying mechanisms by which species diversity drives speciation and the latitudinal gradient of taxonomic groups as species diversity decreases with latitude [4, 5]. Further, in-depth understanding of the genetic and evolutionary mechanisms of speciation genes would not only provide important insights into an organism’s fitness and/or reproduction but also promote conservation of threatened mammalian species through genetic re-engineering, a technique that has recently been used to reverse hybrid sterility in mice by editing the zinc fingers (ZFs) of a widely known speciation gene, PRDM9 [6]. This landmark experiment further signified the important role of PRDM9 in fertility and reproductive compatibility [6]. Nevertheless, the reports of genome-wide non-random distributions of DNA binding motifs and the corresponding clustering of meiotic recombination hotspots, together with the Red Queen model of evolution of these DNA-binding motifs provide convincing evidence of the dominant role of PRDM9 in metazoan speciation [7–25]. Red Queen Hypothesis, which is based on the metaphors in Lewis Carroll’s “Through the Looking Glass” [26], was first used by VanValen [27] to explain speciation dynamics and extinction of species. Since then this metaphor has been widely used as the key hypothesis to test the continual adaptation of species in order to survive in the face of competition and changing environment, including the evolution of ZFs of PRDM9 by treating PRDM9 ZFs as “species” and genome background as “environment” [16, 25]. Nevertheless, the absence of functional PRDM9 in canids [28–30] and presence of single copies of PRDM9 in rodents but multiple copies (i.e., PRDM 7/9) in primates, ruminants and other metazoan lineages [31–33] indicate an interesting yet complex evolutionary history of the PRDM9 gene family.

PRDM9 has been reported to play a dominant role in meiotic recombination in a wide range of mammalian groups [8–10, 13–18, 20, 21, 23, 34–37]. It is a member of the PRDM gene family [33] and encodes a protein with a KRAB, a SSXRD, a PR/SET histone H3(K4) trimethyl transferase domain and a DNA-binding domain consisting of a variable-sized tandemly repeated array of C2H2 ZFs at the C-terminal [18]. The C-terminal array of the C2H2 ZFs domain possesses a DNA-binding function, shows a high diversity and fast evolutionary rate, and hence is likely to have evolved extremely rapidly by positive Darwinian selection [16, 21, 25, 38–40]. However, the N-terminal KRAB, SSXRD and SET domains have evolved at a very slow rate [18], thus making it an ideal genetic marker to trace the evolutionary history of PRDM9 in each metazoan lineage.

Despite the critical role of the PRDM gene family in early development and reproduction [41] little is known about the evolutionary history of these genes. Two recent studies [31, 33] reported the evolution of PRDM gene family and suggested that while primate PRDM9 has a higher similarity of gene structure and protein domain organization with the non-primate co-orthologs and likely retains the features of the ancestral locus, PRDM7 appears to be primate-specific and may have undergone major structural arrangements that decreased the number of ZFs [31]. Vervoort et al. [33] reported that PRDM7 and PRDM9 gene trees do not form separate monophyletic groups and these gene trees are highly incongruent with the species tree, suggesting an unusual evolution of these genes in primates. Further, those studies concluded that PRDM7/9 phylogenetic analysis may be unreliable for positioning the duplication events that have occurred in the primate lineage [33]. Given such unusual evolutionary patterns of PRDM7/9, in particular a non-monophyletic grouping of PRDM9 and PRDM7 in primates [31, 33], one might speculate that PRDM9 and PRDM7 have evolved independently in different metazoan lineages. Therefore, it is unclear if these form monophyletic groups in other metazoan, and we might need to revise the nomenclature of these gene copies.

Utilizing the N-terminal portion of the PRDM9 nucleotide and protein sequences the objective of this study is to investigate the origin and evolution of the multiple copies of PRDM9 in ruminants, to determine the phylogenetic congruencies of gene trees from these novel gene copies with the ruminant species tree, and to assess the underlying genetic and evolutionary forces that shaped the evolution of these gene copies in ruminants. Furthermore, given the fact that each functional domain of the PRDM9 gene is associated with different functions [18], these functional domains are expected to show different evolutionary trajectories. Thus, another objective of this study is to unravel the different evolutionary forces that shape the evolution of N-terminal and that are responsible for a variable-sized tandem-repeat array of C2H2 ZFs at the C-terminal in each lineage. Finally, we propose a model that explains the evolution of PRDM9 and its multiple copies in the ruminant species.

## Results

**Fig. 1:**
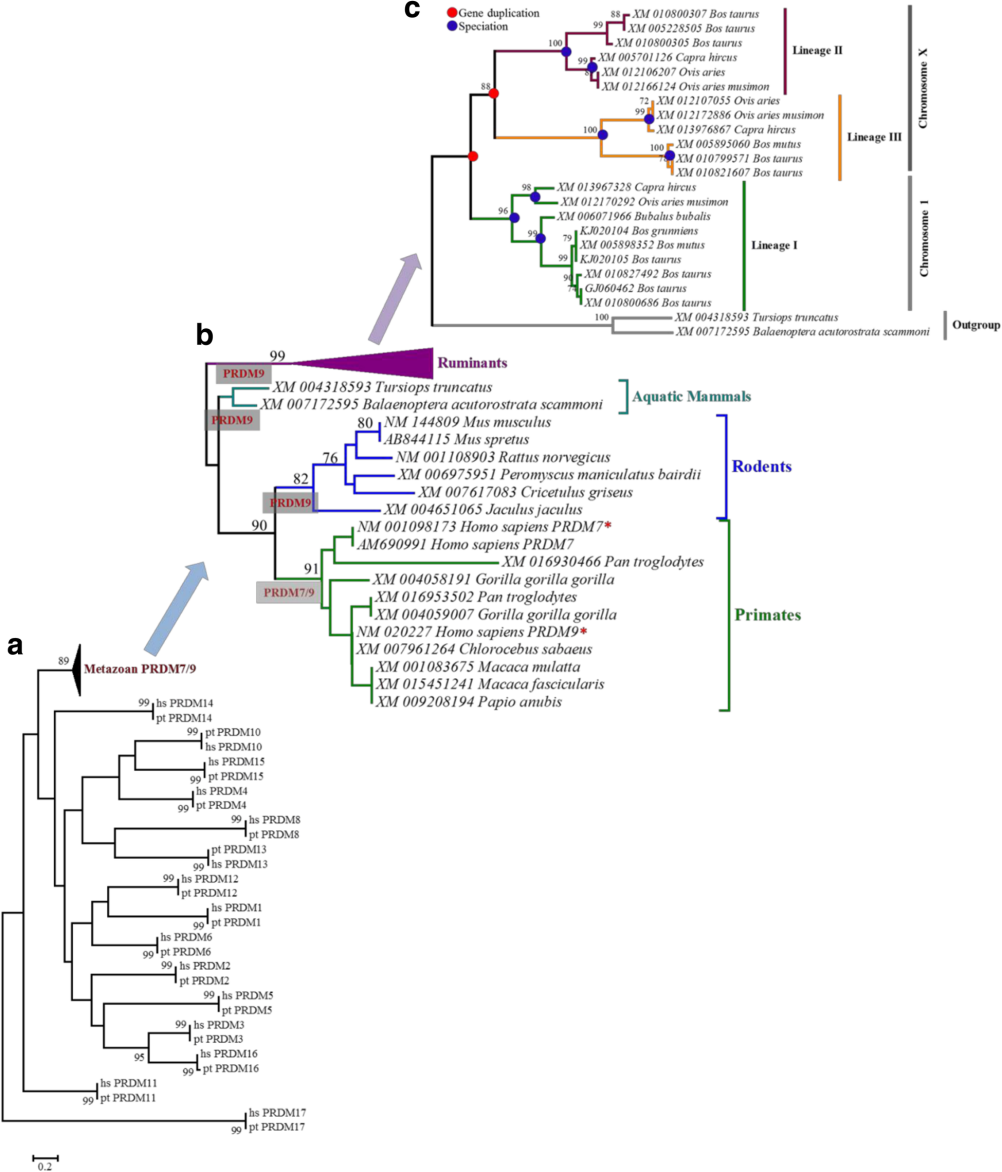
Phylogenetic trees inferred from PRDM genes. **a** Phylogenetic tree inferred from the SET domain amino acid sequences (alignment length: 203 aa) of the PRDM genes. **b** PRDM7/9 gene tree inferred from the SET domain (alignment length: 118 aa). **c** Phylogenetic tree inferred from the N-terminal portion of the amino acid sequences (alignment length: 351 aa) of PRDM9 depicting duplication and speciation events in ruminants. Bootstrap values greater than 70 are shown at the base of the nodes. GenBank accession numbers and scientific names of the species are shown. SET domain sequences representing all the 17 PRDM genes that were previously reported by Fumasoni et al. [31] were used as reference sequences. Asterisks (*) indicate reference sequences for human PRDM7 [52] and human PRDM9 [9, 10]. *hs*: *Homo sapiens; pt: Pan troglodytes*

**Fig. 2:**
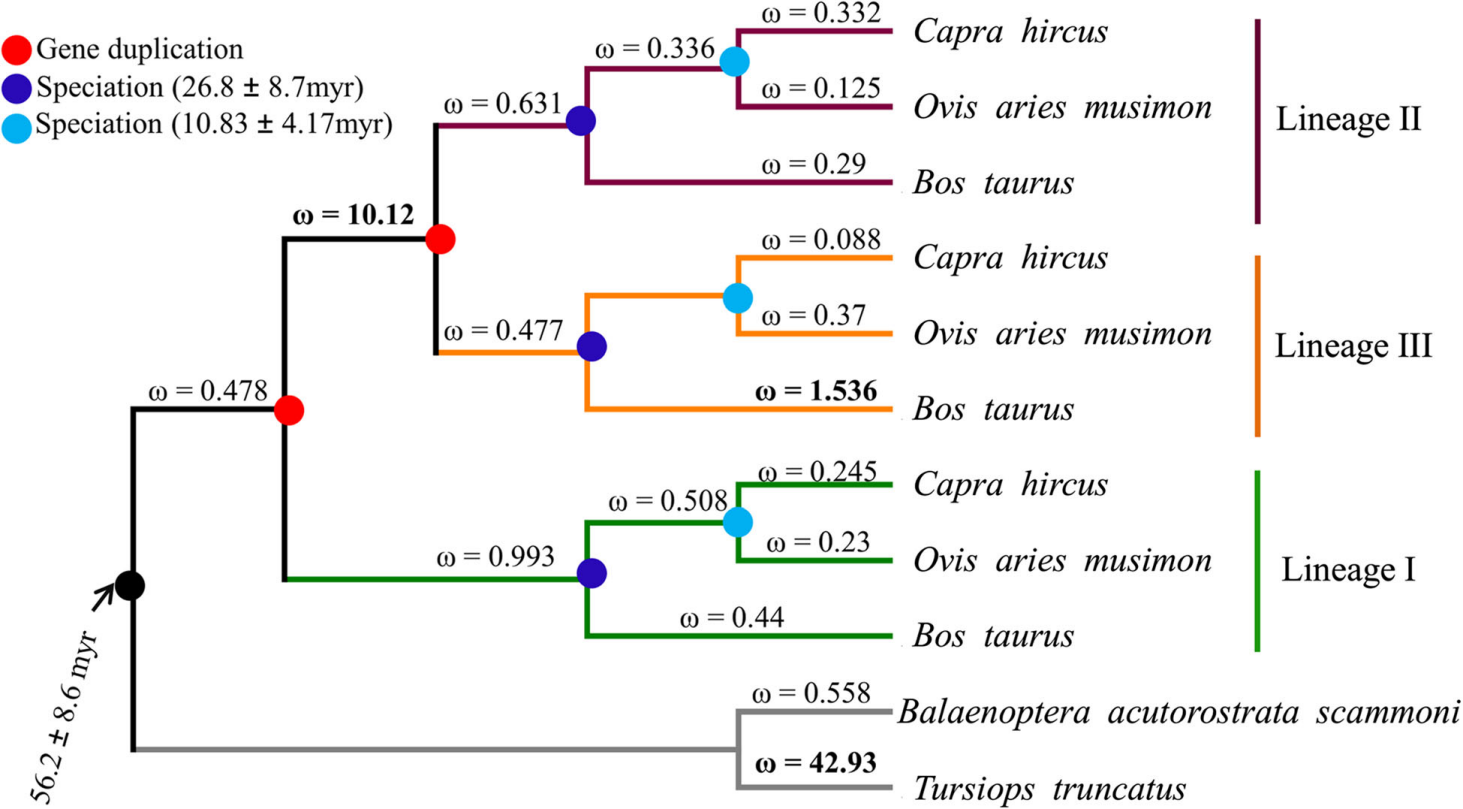
The rate of nonsynonymous (dN) to synonymous (dS) substitutions (ω =;dN/dS) of different branches. The analysis was based on the N-terminal portion of the coding nucleotide sequences of PRDM9. The free-ratio model (M1), which assumes independent ω for each branch, is the best-fit model (*p* =;0.1) over the one-ratio model (M0) that assumes uniform ω for all the branches in the phylogeny. ω >1 are in bold

**Fig. 3:**
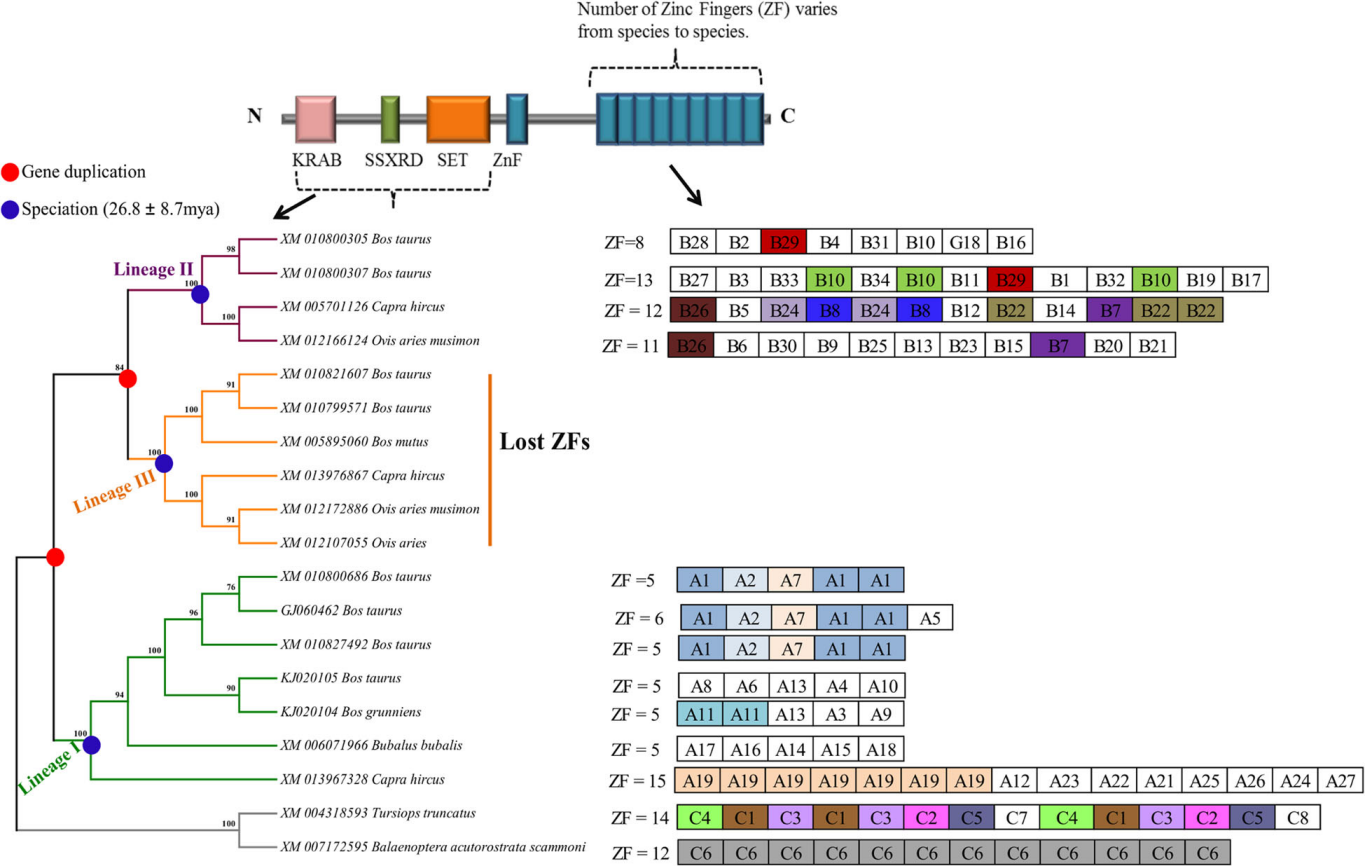
Cladogram and the ZF array arrangements for lineage I, II, and outgroups. Schematic gene (protein coding) structure of the PRDM9 is shown. Approximate locations of each functional domain are identified based on the previous report [18]. Each array is 84-nucleotide base pairs (i.e., 28 AA base pairs). The number of ZFs for each species/individual is mentioned. Each unique array for the respective lineages are shown (Lineage I: A1-A27; Lineage II: B1-B34; Outgroup: C1 - C8). Identical arrays (100% identity at nucleotide level) are color coded. Lineage II showed more number of inter-species identical arrays than any other groups. All groups showed re-arrangements of the arrays within each individual

**Fig. 4:**
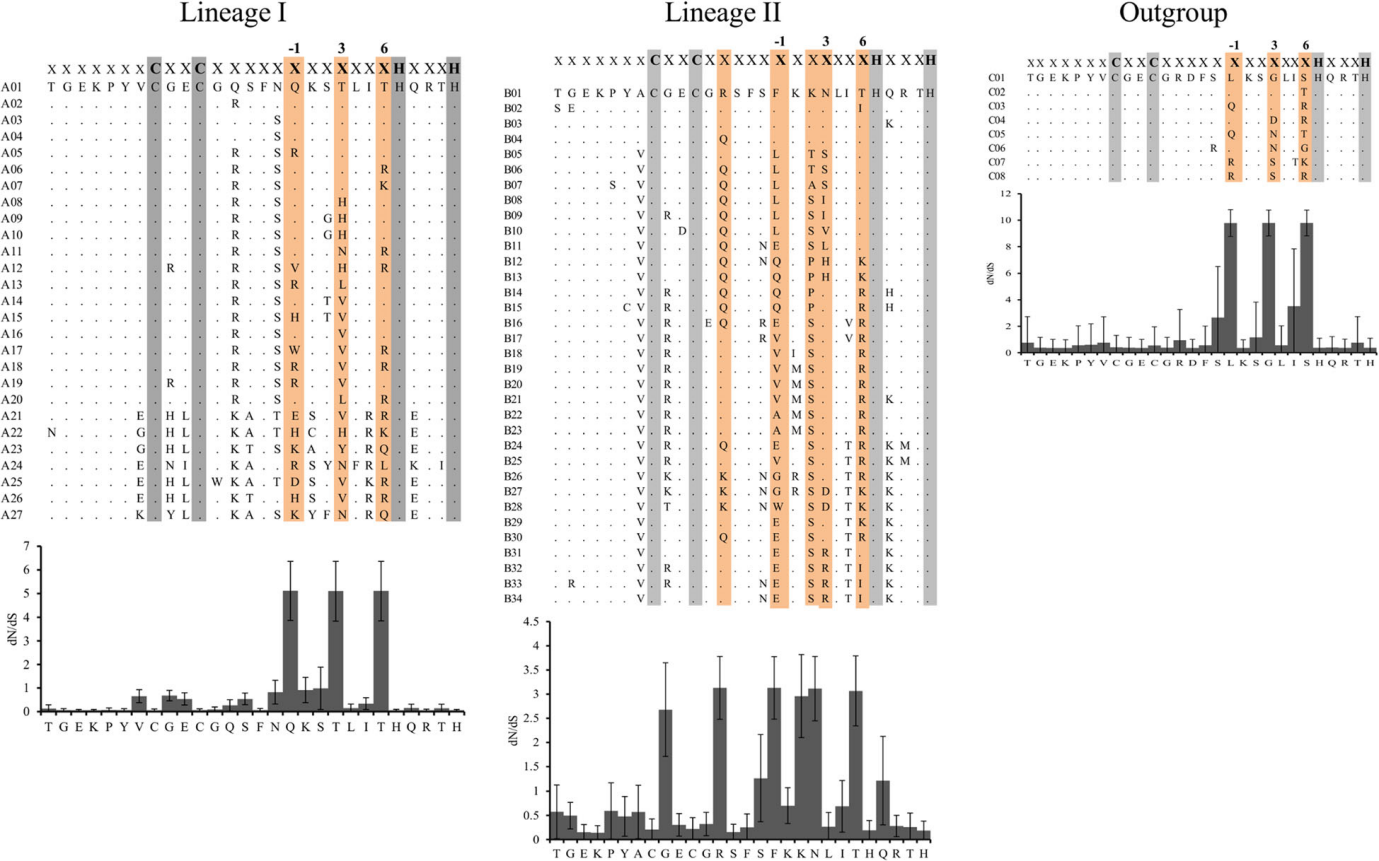
ZF arrays for different lineages and positively selected sites. Sites that are detected to be under positive selection with BEB>0.95 are highlighted in light orange color. C2H2 signature in each lineage is highlighted in grey color. dN/dS for each site in the respective datasets is shown

**Fig. 5:**
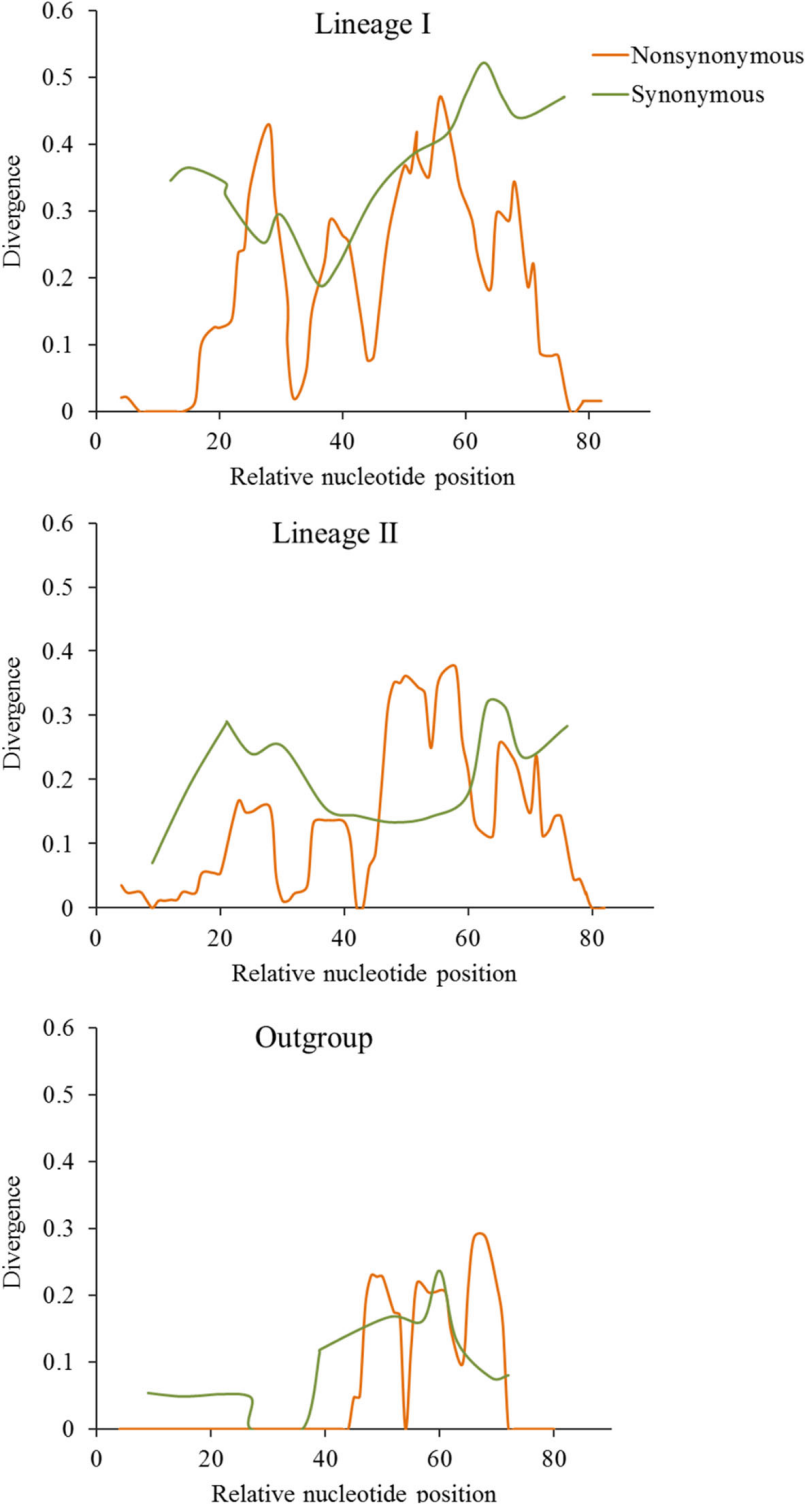
Divergence at the synonymous and nonsynonymous sites. Sliding Window Analyses (SWA) showing the divergence at the synonymous and nonsynonymous sites in the ZF array that comprises 84 nucleotide base pairs for different lineages

**Fig. 6:**
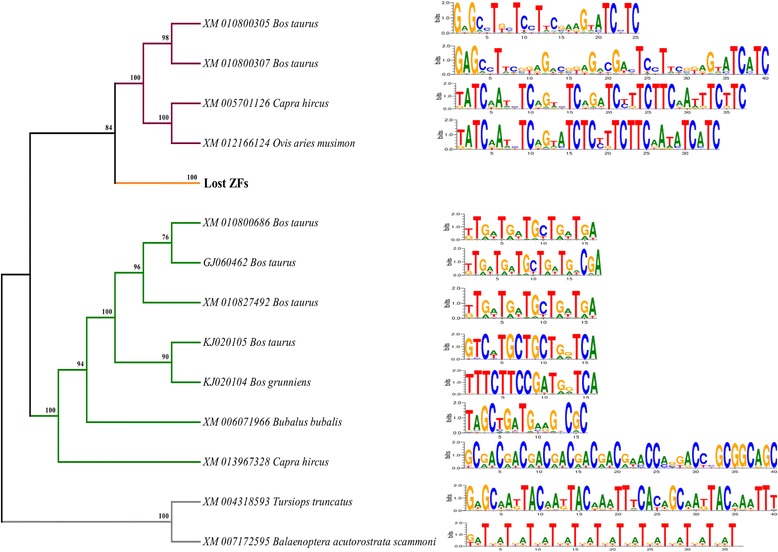
Binding motifs for species representing different lineages

We first give an overview of the main results and then provide more detailed explorations in the following paragraphs. The present study reports (1) evidence of ruminant-specific multiple gene duplication events which likely have had occurred before the ruminant speciation events and after the ancestral ruminant population diverged from its most recent common ancestor (Figs. 1 and 2), (2) the presence of three copies of PRDM9 (Figs. 1 and 2), two copies (lineage II and III; Fig. 1) in chromosome X (chrX) and one copy (lineage I; Fig. 1) in chromosome 1 (chr1) with variable-sized tandemly repeated arrays of C2H2 ZFs at the C-terminal (Fig. 3) thus indicating the possibility of ancient inter- and intra-chromosomal unequal crossing over and gene conversion events, (3) while lineages I and II are characterized by the presence of variable tandemly repeated C2H2 ZFs arrays, lineage III lost these arrays (Fig. 3), (4) C2H2 ZFs of lineages I and II, particularly amino acid residues located at positions −1, 3, and 6 have likely evolved under strong positive selection (Fig. 4; Table 1) thus supporting the notion of previously established Red Queen hypothesis [16, 25], and finally, (5) together with the evidence of positive selection (Fig. 4 Table 1) relatively higher diversities at the nonsynonymous sites (Fig. 5) the presence of identical arrays yet located at different alignment positions in the sister-species (Fig. 3) as well as the observation of variable length of binding motifs for each ruminant species (Fig. 6) support both the concerted evolution [16] and a cyclical back-and-forth evolution of C2H2 ZFs arrays throughout the ruminant evolution spanning millions of years regardless of positive frequency-dependent or negative frequency-dependent selection, a dynamic evolutionary pattern that was recently proposed for host-parasite co-evolution [42, 43].

**Table 1:** Tests for positive selection for the ZFs of each lineage

	Lineage I		Lineage II		Outgroup	
Model Comparison	*2*Δ*l*	*p*	*2*Δ*l*	*p*	*2*Δ*l*	*p*
M1a vs M2a	20.1377	0.000042	13.8381	0.00099	22.9932	0.000010
M7 vs M8	20.4410	0.000036	17.5405	0.00016	23.1771	0.000009
M8 vs M8a	18.3856	0.000018	12.9539	0.00032	22.9906	0.000002

Neutral (null) models: M1a, M7, M8a

Selection (alternative) models: M2a, M8

Degrees of freedom: 2 for M1a-M2a, M7-M8; and 1 for M8-M8a

*Δl*: Differences between the likelihood scores of null and alternative models

To evaluate the phylogenetic positioning (Fig. 1a) and clustering (Fig. 1b) of PRDM7 and PRDM9 in the PRDM7/9 gene tree and to assess the evolutionary origin of multiple copies of PRDM9 in ruminants (Fig. 1c) we reconstructed the phylogenetic trees using the amino acid sequences of the PR domains located at the N-terminal region (Fig. 1a b, and c). Consistent with previous studies [31, 33], our analyses revealed that PRDM7/9 form unique clusters (Fig. 1a) and that PRDM7 is primate-specific (Fig. 1b). The N-terminal amino acid sequence-based phylogeny showed that each PRDM9 copy (lineage I-III) of ruminants formed a separate monophyletic group and showed the evidence of two gene duplication events prior to the ruminant speciation (Fig. 1c). Multiple paralog copies of PRDM9 in ruminants (e.g., genus: *Bos*, *Capra*, and *Ovis*) support gene duplications before the speciation events. Based on the previous reports [44–47], these three species (genus: *Bos*, *Capra*, and *Ovis*) had a shared ancestry. *Bos* diverged from the common ancestral population approximately 26.8 (±8.7) million years ago (mya), and the split between *Capra* and *Ovis* was estimated to be approximately 10.83 (±4.17) mya. Concurrently, the presence of all three PRDM9 copies in each species provides strong evidence of the gene duplication events before ruminant speciation (i.e., 26.8 ± 8.7 mya) (Fig. 2).

**Fig. 7:**
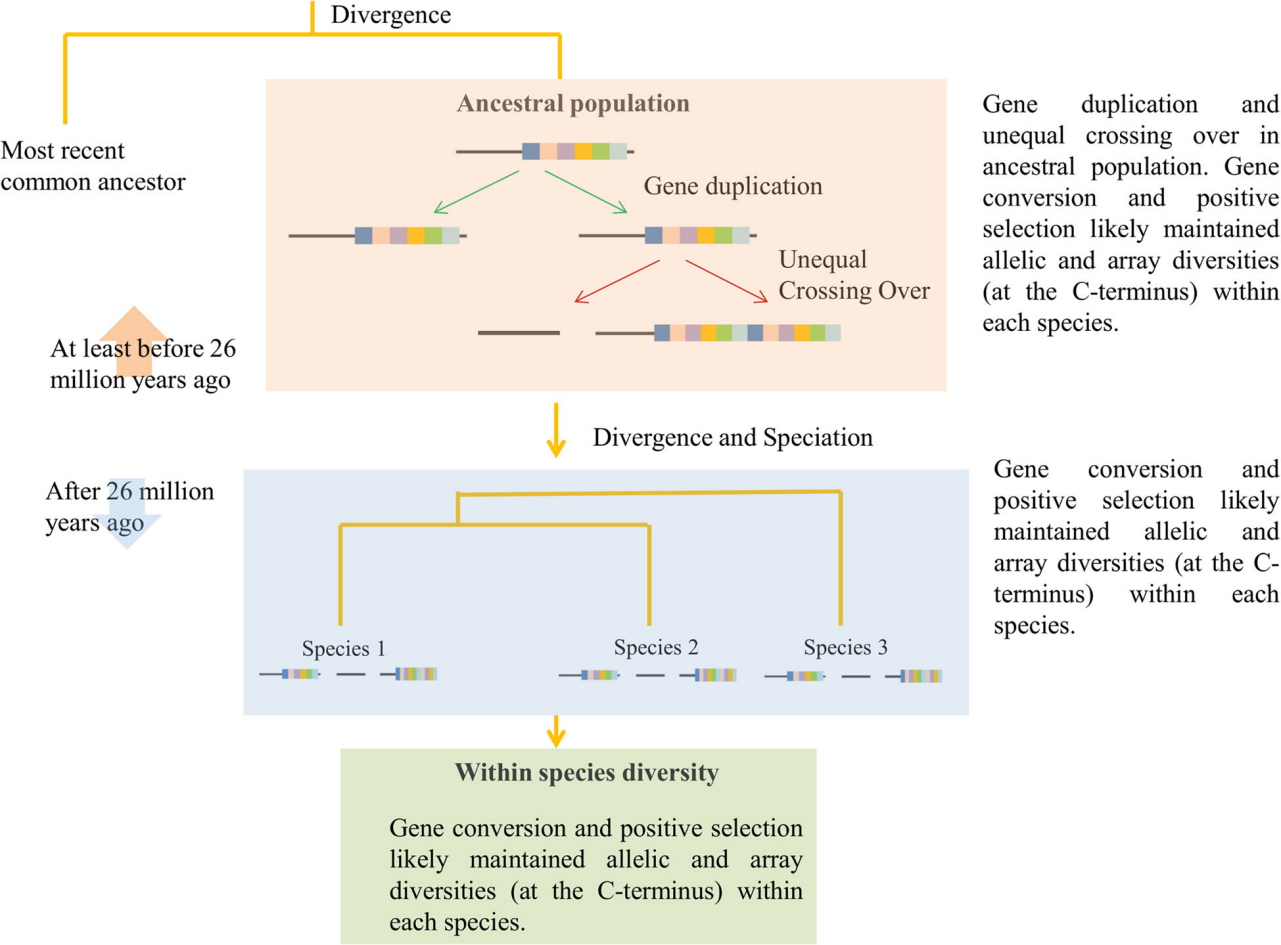
Proposed model of PRDM9 evolution in ruminants. The proposed model is based on the evidence of gene duplication, inter-species and within species gene conversion, speciation and divergence time, as well as the evidence of variable selection pressures at the N- and C-terminal regions of the PRDM9

One of the striking observations is the presence of two copies of PRDM9 (lineage II and III) on chrX (Fig. 1). While one X-linked copy is characterized by the presence of variable-sized tandemly repeated C2H2 ZFs (lineage II) the other copy completely lost its ZFs (lineage III). Interestingly, the dN/dS ratio (ω), for the branch leading to X-linked lineages (i.e., II and III) was estimated to be 10.12, indicating the evidence of positive selection, a typical characteristic of novel gene copies after a duplication event [48, 49]. We also found that the C-terminal C2H2 ZFs of lineages I and II and the outgroup, especially the amino acid residues at the positions −1, 3 and 6 that played crucial roles in DNA binding during meiotic recombination [16], have likely evolved under strong positive selection (Fig. 4). The tandemly repeated arrangement of the ZFs and the presence of identical ZFs (for example, in lineage I: A1, A7, A11 and in lineage II: B7, B8, B10, B22, B26, and B29) showed evidence of concerted evolution of C2H2 ZFs of both X-linked (lineage II) and autosomal (lineages I) PRDM9 copies (Fig. 3). Further, we observed species-specific, lineage-specific, and individual-level variations of the length of tandemly repeated C2H2 ZFs as well as variations in the predicted binding motifs (Fig. 6). Finally, taking all the possible evolutionary forces (e.g., concerted evolution, gene duplications, and positive selection) that likely affected the evolution of PRDM9 and maintained genetic variations even at the individual levels in these economically important ruminant species (genus: *Bos*, *Capra*, and *Ovis*) into consideration, we presented a schematic model to describe how the multiple copies of PRDM9 are derived and evolved in the ruminant species (Fig. 7).

## Discussion

Despite the fact that PRDM9 is a key regulator of meiotic recombination [7–18, 20, 21, 34, 35, 37, 50, 51] and plays a dominant role in hybrid incompatibility [6], little is known about the underlying genetic and evolutionary mechanisms that generated multiple copies of PRDM9 in many metazoan lineages. The present study elucidates the underlying evolutionary genetic mechanisms that shaped the evolution of PRDM9, an important speciation gene [16, 18], in the economically important ruminants species (genus *Bos*, *Capra*, and *Ovis*). These domesticated ruminants are estimated to have diverged from a common ancestor approximately 26.8 (±8.7) mya [44–47]. In contrast to primate’s PRDM7 and PRDM9 gene copies that form non-separate monophyletic groups and show ambiguities concerning the phylogenetic positioning of the gene duplication events in the primate phylogeny [33], the observation of deep-split among the three lineages together with a strong statistical support for monophyletic groups provide convincing evidence of two gene duplication events before the ruminant speciation. Taken together with the results of a previous study [33], our study suggests that the PRDM9 duplication event in ruminants, which is estimated to have had occurred sometime between 27 and 56 mya, is ruminant-specific and likely occurred after the split of the ruminants ancestral populations from the most recent common ancestor. Nevertheless, based on these results, one might speculate that PRDM9 of other mammalian lineages may also exhibit unique phylogenetic histories. Further, together with the results of a previous study [33], we ascertained that the primate-specific PRDM7 [31, 33, 52] is not phylogenetically closely related with the novel copies of ruminant PRDM9, therefore, warrants separate nomenclature of PRDM9 copies belonging to lineage II and III.

Although gene duplication events through inter-chromosomal especially, autosomal crossing-overs are common across the mammalian groups [53], the observations of gene duplications between sex chromosomes and autosomes is a unique event. Interestingly, a previous study has also reported inter-chromosomal duplications of the adrenoleukodystrophy (ADL) locus from chrX to chromosomes 2p11, 10p11, 16p11 and 22q11 in humans [54]. However, to our knowledge, so far no such inter-chromosomal duplications between chrX and autosomes have been reported for any other mammalian taxa. We previously found a strong association between PRDM9 on chr1 and recombination phenotypes in cattle [13]. Sandor et al. [19] have also reported the presence of an X-linked PRDM9 and have detected several polymorphisms in the corresponding C2H2 ZFs. Although PRDM9 is present on both chr1 and chrX in cattle, the genetic and evolutionary mechanisms of the evolution of PRDM9 on the two chromosomes remain unclear. The presence of X-linked PRDM9 copies in ruminants could possibly be a rare event and be explained by some unique evolutionary mechanisms. Sex chromosomes were derived from a pair of ancestral autosomes [55] and have evolved independently many times during the mammalian evolution [56]. Additionally, Ohta [57] proposed that inter-and intra-chromosomal unequal crossing overs, coupled with mutation and random drift, are among the fundamental forces in the evolution of multigene families. More importantly, inter-and intra-chromosomal unequal crossing overs have been shown to have a dominant effect on the contraction and expansion of genes in a given family [57, 58]. Therefore, it could be possible that the ancestral locus of PRDM9, which is originally located at the autosomal region in most of the metazoans, appeared in the ruminant’s chrX through unequal crossing overs, which might have happened millions of years ago possibly prior to ruminant's speciation and resulted in two additional copies of X-linked PRDM9. Given the fact that ruminants PRDM9 copies have been in the autosome and in the X chromosome for at least the past 27 million years, these copies are predicted to have differential evolutionary trajectories [56]. Mammalian sex chromosome genes are predicted to evolve at a much higher rate, and the fixation rate of beneficial mutations is predicted to be higher for X-linked genes than that of autosomal genes [56]. Interestingly, the observed elevated dN/dS ratio (i.e., dN/dS > 1), which indicates the evidence of positive selection, further supports the notion of accelerated rate of evolution of novel gene copies after a duplication event [48, 49]. Additionally, these duplicated copies may also have some functional consequences, and three possibilities would be expected [48, 59, 60]: i) the novel copies are likely to have experienced relaxed selection pressure and ultimately may acquire deleterious mutations that lead to loss of function, a process known as non-functionalization; ii) in rare cases the novel copies can acquire beneficial mutations that differentiate their functions from that of the ancestor, a process known as neo-functionalization; and iii) mutations may occur in both ancestor and duplicated copies of a gene and result in complementary functions which is known as sub-functionalization [59, 60]. The presence of a stop-codon at the KRAB region in three sequences representing the genus *Ovis* and *Capra* of the lineage III (Additional file 1) supports the notion of non-functionalization; however an artifact of sequencing errors cannot be ruled out.

Although it is apparent that PRDM9 of chr1 regulates meiotic recombination in cattle [13] the functional significance of the X-linked PRDM9 is yet to be explored. Nevertheless, even in the absence of gene duplication event, sex chromosome genes are predicted to evolve at a faster rate than autosomal genes [56]. Therefore, the mutation rate of the X-linked PRDM9 is expected to be higher than that of the autosomal copy. However, due to the limited sample size, we could not directly estimate the mutation rate for each lineage, but the observation of incomplete lineage sorting for *Bos* species in chr1 may be an indication of slower mutation rate of lineage I. This inference, however, should be taken with caution since sequences representing more species are required to test the hypothesis of mutational differences between the X-linked and autosomal PRDM9 copies.

In contrast to the N-terminal portion of PRDM9 which comprises three conserved functional domains [18], the C-terminal C2H2 ZFs of lineages I and II and the outgroup, especially the amino acid residues at the positions −1, 3 and 6 that played crucial roles in DNA binding during meiotic recombination [16], have likely evolved under strong positive selection. Although this observation of extremely rapid evolution of ruminant’s PRDM9 C2H2 ZFs by positive Darwinian selection is nothing surprising and has been reported for several other mammalian species [16, 21, 32, 38, 39], the evidence of positive selection on the X-linked C2H2 ZFs is one of the most striking observations. This compelling evidence of positive selection on the X-linked C2H2 ZFs PRDM9 indicates some unknown functional significance, thus warrants further investigation on the functional significance of the X-linked PRDM9 C2H2 ZFs. Consistent with a previous study [16], the present study has also showed evidence of concerted evolution of both X-linked (lineage II) and autosomal (lineages I) ZFs of PRDM9, which explained the species-specific, even at the individual level, variations in the length of the tandemly repeated C2H2 ZFs and the predicted binding motifs as well.

## Conclusions

In stark contrast to the primate lineage where the PRDM9 duplication mechanism is still an unresolved issue [33] our study provides strong evidence that the autosomal PRDM9 of ruminants has been duplicated to the X chromosome in the ruminants, which likely happened before the ruminant speciation events. The presence of X-linked PRDM9 copies in ruminants could possibly be a rare event and may be explained by some unique evolutionary mechanisms, possibly, through unequal crossing-overs. Nevertheless, the inter-chromosomal duplications before the ruminant’s speciation together with the persistent positive selection and concerted evolution of ZFs, at both species and individual levels, shaped the evolution of autosomal and X-linked PRDM9 in ruminants. Collectively, this study reports the unique evolutionary mechanism of PRDM9 in ruminants, including the presence of duplicated copies of PRDM9 on chr1 and chrX both with active C2H2 ZFs under positive selection. Concomitantly, a recent study has also reported extensive diversity of PRDM9 in several ruminant species [40]. Nevertheless, given such lineage-based unique evolutionary trajectories of the PRDM9, as demonstrated in the present study as well as in previous studies (eg., [16, 33]), taking more taxonomic lineages into consideration, future studies should be carried out to unravel the evolutionary trajectory of this important speciation gene across the metazoans.

## Methods

### Phylogenetic analyses

To unravel the evolutionary dynamics of PRDM9 and its novel copies in ruminants using the previously characterized cattle PRDM9 as reference sequences ([61] GenBank accession numbers: GJ060462 KJ020105), all the available complete coding nucleotide sequences of ruminant PRDM9 were retrieved from GenBank [62] (Additional file 4). Since the PRDM gene sequences have varying numbers of Zinc Finger (ZF) repeat sequences at their C-terminal domain, to avoid non-specific hits, we used the N-terminal portion of the PRDM amino acid sequences of the reference genomes and subsequently retrieved the complete DNA sequence of each PRDM7/9. Using the well-characterized and annotated human [9, 10, 52], mouse [32], and cattle [61] PRDM 7/9 protein sequences, we also retrieved the PRDM7/9 protein sequences representing primates, rodents, ruminants, and aquatic mammalian groups from GenBank. The conserved SET domain that comprises 118 amino acids was used for phylogenetic reconstruction of PRDM7/9 gene tree and specifically to assess the phylogenetic positioning of the human PRDM7. We aligned the protein sequences and manually checked the sequence quality using MEGA7 [63]. Amino acid alignments of the N-terminal functional domains representing different taxonomic groups (primates, rodents, ruminants, and aquatic mammals) are shown in Additional files 1, 2 and 3. To reconstruct the PRDM gene tree, amino acid sequences of the SET domain of 17 PRDM genes [31] were used as reference sequences. Based on the previous reports, functional domains of PRDM9 were identified [16, 18]. Sequences were aligned using the MUSCLE algorithm implemented in MEGA7 [63]. All the sequences were manually visualized to ensure high quality. Since the N-terminal portion of the PRDM9 that comprises three functional domains has slower evolutionary rate and evolutionarily conserved across the metazoan lineages [18], we used this portion of the sequences to infer evolutionary history and phylogenetic relatedness among the novel copies of the ruminant’s PRDM9. Protein alignment of the N-terminal portion of the PRDM9 and its novel copies are shown in Additional files 1 and 3. Aquatic mammals seemed to have close phylogenetic affiliation with ruminants [46], therefore PRDM9 of aquatic mammals were used as outgroup. Nucleotide and amino acid based maximum-likelihood (ML) phylogenies were reconstructed under appropriate substitution models in MEGA7 [63]. Appropriate models of nucleotide and amino acid substitutions for the respective datasets were selected under the Bayesian Information Criterion (BIC) implemented in MEGA7. JTT (Jones–Taylor–Thornton) + G (gamma distribution shape parameter) [64] and TrN93 (Tamura-Nei) + G [65], respectively, were the best-fit amino acid and nucleotide substitution models selected by BIC. Using the same program, nodal supports were estimated with 1000 bootstrap replicates. The time of divergence of the respective clades/species that were previously estimated based on the fossil based molecular clock calibration [44–47] were used to determine the timing of ruminant’s speciation and ruminant’s PRDM9 duplication events. The ZF arrays in each PRDM9 sequences were identified according to the previously defined nomenclature [9]. The putative DNA binding motifs for each PRDM9 C2H2 were predicted using the software (available at: http://compbio.cs.princeton.edu/zf/) [66, 67], which has been previously used in the prediction of PRDM9 binding motifs in primates [14, 68, 69].

### Test for positive selection

Given the fact that the presence of recombinant sequences in the data set could potentially affect the selection analyses [70, 71] using the recombination detection programs (RDP) implemented in RDP ver. 3 [72], we performed recombination detection analyses to ensure there are no recombinant sequences in the respective data sets used in selection analyses. The ratio of nonsynonymous (dN) to synonymous (dS) substitutions (ω = dN/dS), which has been widely used to measure the strength of selection on a protein-coding gene [73, 74], was used to measure the selection pressures in each dataset under five codon-based substitution models (neutral models: M1a, M7, M8a; selection models: M2a M8) that are implemented in the CODEML of the PAML 4.7 package [75], and their performances were evaluated using Likelihood Ratio Tests (LRTs) [73, 76]. Codon sites with Bayes-Empirical Bayes (BEB) posterior probability ≥ 0.95 were considered to be under positive selection. The inferred unrooted ML trees for the respective datasets that were used as input trees for the CODEML program were reconstructed using the PhyML ver. 3 [77]. To know whether ω varies across the branches, using the inferred phylogeny we compared the free-ratios model (M1), which assumes an independent ω for each branch, with the one-ratio model (M0) that assume uniform ω across the branches [76]. LRT was used to select the best-fit model. To check consistency of the selection results, we performed selection analyses using different input trees that were built under different tree-building methods implemented in MEGA [63] and PhyML [77]. Our selection results are very much consistent and are not biased by different tree building methods. To know the patterns of nonsynonymous and synonymous variations across the ZFs in respective lineages, using the DNAsp ver 5.0 [78], we also performed Sliding Window (window length = 5bp step size = 1bp) Analyses (SWA).

## Additional files

Additional file 1: Amino acid alignment of the N-terminal region of the PRDM9. (PDF 187 kb)
Additional file 2: Amino acid alignment of the SET domain of PRDM7/9. (PDF 100 kb)
Additional file 3: The PR domains of human PRDM7 and PRDM9 are aligned with the corresponding sequences of each lineage. (PDF 96 kb)
Additional file 4: GenBank accession numbers of the PRDM9 nucleotide sequences analyzed in this study. (PDF 80 kb)

## Abbreviations

BDM: Bateson-Dobzhansky-Muller
BIC: Bayesian information criterion
dN: Nonsynonymous
dS: Synonymous
JTT: Jones–Taylor–Thornton
LRT: Likelihood ratio test
ML: Maximum-likelihood
PRDM9: PR domain9
SWA: Sliding window analyses
TrN93: Tamura-Nei
ZFs: Zinc fingers
