# Supplementary material for: Ruminant-specific multiple duplication events of PRDM9 before speciation
Source: BMC Evol Biol. 2017 Mar 14;17:79. doi: 10.1186/s12862-017-0892-4 (PMC5351255; doi:10.1186/s12862-017-0892-4)
Supplement: Additional file 1: — Amino acid alignment of the N-terminal region of the PRDM9. (PDF 187 kb) [file 12862_2017_892_MOESM1_ESM.pdf]

Additional file 1. Amino acid alignment used in the phylogenetic analyses. Lineages are color coded. Amino acids unique to each lineage are colored coded. Three stop codons that were detected in KRAB region of three species belong to lineage III are highlighted in red. These stop codons were replaced with gaps (-) for selection analyses. Dot (.) indicates identical amino acid residue.

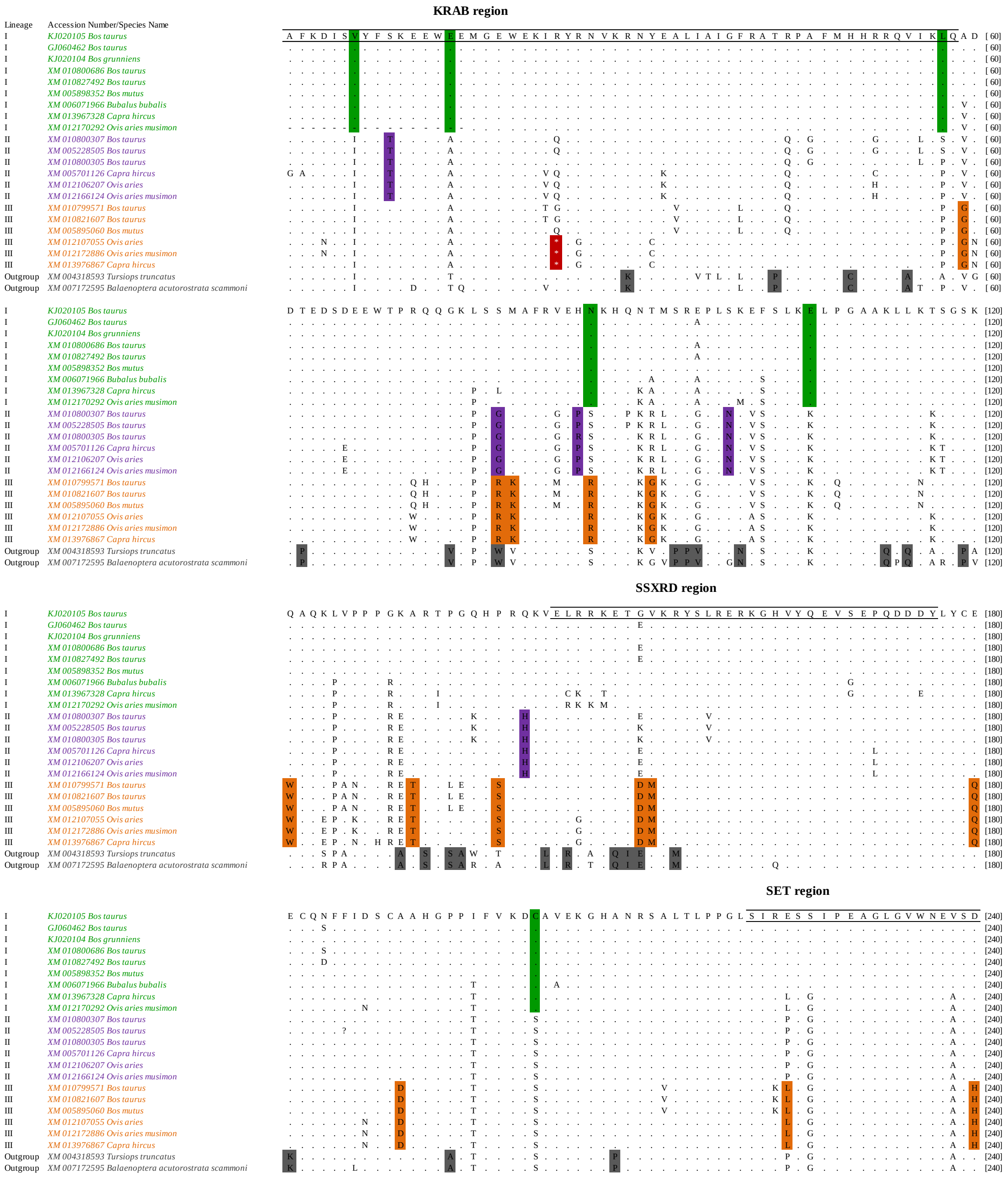

Cont….

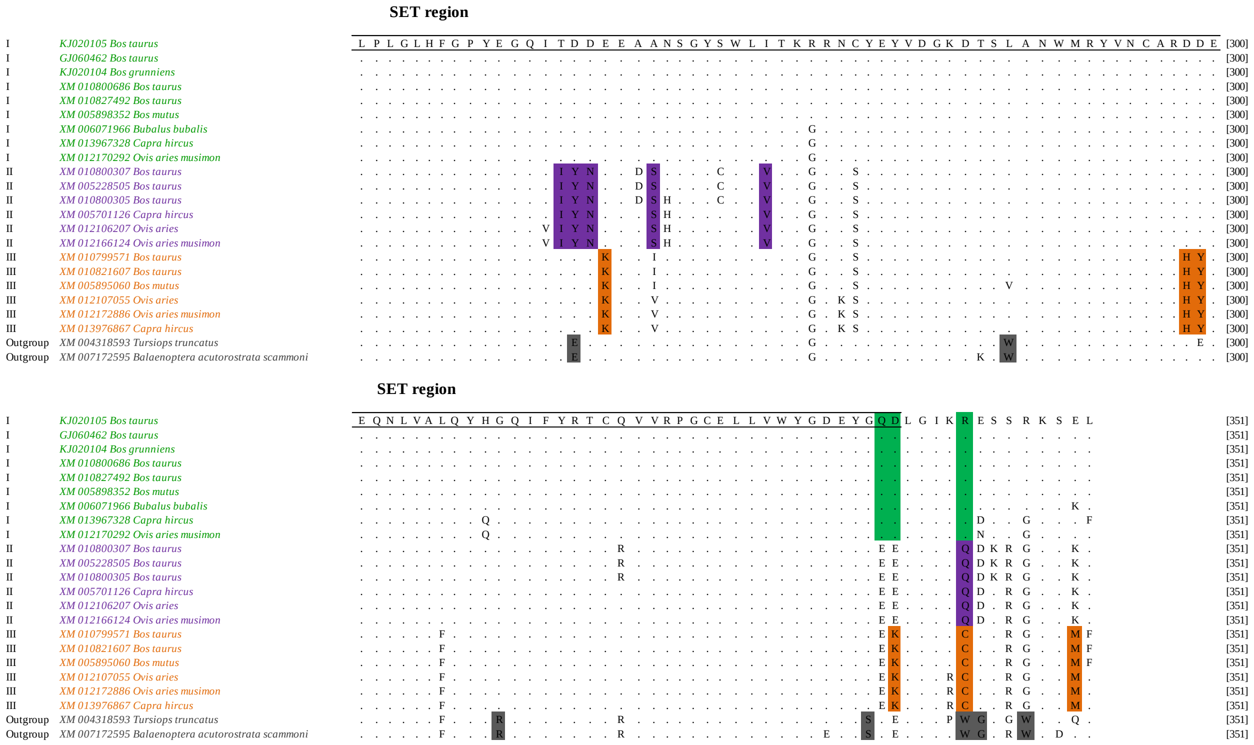
SET region
L P L G L H F G P Y E G Q I T D D E E A A N S G Y S W L I T K R R N C Y E Y V D G K D T S L A N W M R Y V N C A R D D E [300]
I KJ020105 Bos taurus
I GJ060462 Bos taurus
I KJ020104 Bos grunniens
I XM 010800686 Bos taurus
I XM 010827492 Bos taurus
I XM 005898352 Bos mutus
I XM 006071966 Bubalus bubalis
I XM 013967328 Capra hircus
I XM 012170292 Ovis aries musimon
II XM 010800307 Bos taurus
II XM 005228505 Bos taurus
II XM 010800305 Bos taurus
II XM 005701126 Capra hircus
II XM 012106207 Ovis aries
II XM 012166124 Ovis aries musimon
III XM 010799571 Bos taurus
III XM 010821607 Bos taurus
III XM 005895060 Bos mutus
III XM 012107055 Ovis aries
III XM 012172886 Ovis aries musimon
III XM 013976867 Capra hircus
Outgroup XM 004318593 Tursiops truncatus
Outgroup XM 007172595 Balaenoptera acutorostrata scammoni
SET region
E Q N L V A L Q Y H G Q I F Y R T C Q V V R P G C E L L V W Y G D E Y G Q D L G I K R E S S R K S E L [351]
