# Supplementary material for: Ruminant-specific multiple duplication events of PRDM9 before speciation
Source: BMC Evol Biol. 2017 Mar 14;17:79. doi: 10.1186/s12862-017-0892-4 (PMC5351255; doi:10.1186/s12862-017-0892-4)
Supplement: Additional file 2: — Amino acid alignment of the SET domain of PRDM7/9. (PDF 100 kb) [file 12862_2017_892_MOESM2_ESM.pdf]

Additional file 2 Alignment of SET domain of PRDM7/9. Dot (.) indicates identical amino acid residue.

	Accession Number/Scientific Name		
Primates	NM_ 020227 *Homo sapiens* PRDM9	RIGPSGIPQAGLGVWNEASDLPLGLHFGPYEGRITEDEEAANNGYSWLITKGRNCYEYVD	[60]
	NM_ 001098173 *Homo sapiens* PRDM7	....S.................	[60]
	AM690991 *Homo sapiens* PRDM7	....S.................	[60]
	XM_ 016953502 *Pan troglodytes*	..............................K.............................	[60]
	XM_ 016930466 *Pan troglodytes*	............R.......P.....S.....Q.........S.................	[60]
	XM_ 001083675 *Macaca mulatta*	...................................Q........................	[60]
	XM_ 015451241 *Macaca fascicularis*	...................................Q........................	[60]
	XM_ 004058191 *Gorilla gorilla gorilla*	.....................................K....S.................	[60]
	XM_ 004059007 *Gorilla gorilla gorilla*	..............................K.............................	[60]
	XM_ 009208194 *Papio anubis*	...................................Q........................	[60]
	XM_ 007961264 *Chlorocebus sabaeus*	....	[60]
Rodents	NM_ 144809 *Mus musculus*	..S.....E.............V.........Q.........S.................	[60]
	AB844115 *Mus spretus*	..S.....E.............V.........Q.........S.................	[60]
	NM_ 001108903 *Rattus norvegicus*	........E.............V.......K.Q.........S.................	[60]
	XM_ 006975951 *Peromyscus maniculatus bairdii*	...S....E.............V.......K.Q.........S................N	[60]
	XM_ 004651065 *Jaculus jaculus*	..R.....D.............V.........Q.........S....M............	[60]
	XM_ 007617083 *Cricetulus griseus*	........E.............V.......K.Q..D......S.................	[60]
Ruminants	KJ020105 *Bos taurus*	S.RE.S..E........V..............Q..D......S........R........	[60]
	GJ060462 *Bos taurus*	S.RE.S..E........V..............Q..D......S........R........	[60]
	KJ020104 *Bos grunniens*	S.RE.S..E........V..............Q..D......S........R........	[60]
	XM_ 010800686 *Bos taurus*	S.RE.S..E........V..............Q..D......S........R........	[60]
	XM_ 010827492 *Bos taurus*	S.RE.S..E........V..............Q..D......S........R........	[60]
	XM_ 005898352 *Bos mutus*	S.RE.S..E........V..............Q..D......S........R........	[60]
	XM_ 006071966 *Bubalus bubalis*	S.RE.S..E........V..............Q..D......S.................	[60]
	XM_ 013967328 *Capra hircus*	S.RL....E.......................Q..D......S.................	[60]
	XM_ 012170292 *Ovis aries musimon*	S.RL....E.......................Q..D......S.................	[60]
	XM_ 010800307 *Bos taurus*	S.R.....E.......................Q.IYN..DS.S..C..V.....S.....	[60]
	XM_ 005701126 *Capra hircus*	S.R.....E.......................Q.IYN...SHS.....V.....S.....	[60]
	XM_ 012106207 *Ovis aries*	S.R.....E.......................QVIYN...SHS.....V.....S.....	[60]
	XM_ 012166124 *Ovis aries musimon*	S.R.....E.......................QVIYN...SHS.....V.....S.....	[60]
	XM_ 010800305 *Bos taurus*	S.R.....E.......................Q.IYN..DSHS..C..V.....S.....	[60]
	XM_ 005228505 *Bos taurus*	S.R.....E.......................Q.IYN..DS.S..C..V.....S.....	[60]
	XM_ 012107055 *Ovis aries*	S.RL....E..........H............Q..D.K..V.S..........KS.....	[60]
	XM_ 005895060 *Bos mutus*	S.KL....E..........H............Q..D.K..I.S...........S.....	[60]
	XM_ 010799571 *Bos taurus*	S.KL....E..........H............Q..D.K..I.S...........S.....	[60]
	XM_ 010821607 *Bos taurus*	S.KL....E..........H............Q..D.K..I.S...........S.....	[60]
	XM_ 012172886 *Ovis aries musimon*	S.RL....E..........H............Q..D.K..V.S..........KS.....	[60]
	XM_ 013976867 *Capra hircus*	S.RL....E..........H............Q..D.K..V.S..........KS.....	[60]
Aquatic mammals	XM_ 004318593 *Tursiops truncatus*	S.R.....E.......................Q.........S.................	[60]
	XM_ 007172595 *Balaenoptera acutorostrata scammoni*	S.R.....E.......................Q.........S.................	[60]

	Accession Number/Scientific Name		
Primates	NM_ 020227 *Homo sapiens* PRDM9	GKDKSWANWMRYVNCARDDEEQNLVAFQYHRQIFYRTCRVIRPGCELLVWYGDEYGQE	[118]
	NM_ 001098173 *Homo sapiens* PRDM7	.....S..S.......	[118]
	AM690991 *Homo sapiens* PRDM7	.....S..S.......	[118]
	XM_ 016953502 *Pan troglodytes*	....	[118]
	XM_ 016930466 *Pan troglodytes*	............E............S......S.................S..K....	[118]
	XM_ 001083675 *Macaca mulatta*	....	[118]
	XM_ 015451241 *Macaca fascicularis*	....	[118]
	XM_ 004058191 *Gorilla gorilla gorilla*	..........................L...............................	[118]
	XM_ 004059007 *Gorilla gorilla gorilla*	....	[118]
	XM_ 009208194 *Papio anubis*	....	[118]
	XM_ 007961264 *Chlorocebus sabaeus*	....	[118]
Rodents	NM_ 144809 *Mus musculus*	.Q.E.Q.........................K.........................	[118]
	AB844115 *Mus spretus*	.Q.E.Q.........................K.........................	[118]
	NM_ 001108903 *Rattus norvegicus*	.Q.E.Q.........................K............R.............	[118]
	XM_ 006975951 *Peromyscus maniculatus bairdii*	.Q.E.Q..	[118]
	XM_ 004651065 *Jaculus jaculus*	...E.Q......................................S.............	[118]
	XM_ 007617083 *Cricetulus griseus*	.QEE.Q...................................N................	[118]
Ruminants	KJ020105 *Bos taurus*	...T.L....................L...G.......Q.V................D	[118]
	GJ060462 *Bos taurus*	...T.L....................L...G.......Q.V................D	[118]
	KJ020104 *Bos grunniens*	...T.L....................L...G.......Q.V................D	[118]
	XM_ 010800686 *Bos taurus*	...T.L....................L...G.......Q.V................D	[118]
	XM_ 010827492 *Bos taurus*	...T.L....................L...G.......Q.V................D	[118]
	XM_ 005898352 *Bos mutus*	...T.L....................L...G.......Q.V................D	[118]
	XM_ 006071966 *Bubalus bubalis*	...T.L....................L...G.......Q.V................D	[118]
	XM_ 013967328 *Capra hircus*	...T.L....................L..QG.......Q.V................D	[118]
	XM_ 012170292 *Ovis aries musimon*	...T.L....................L..QG.......Q.V................D	[118]
	XM_ 010800307 *Bos taurus*	...T.L....................L...G.........V...............E.	[118]
	XM_ 005701126 *Capra hircus*	...T.L....................L...G.......Q.V...............E.	[118]
	XM_ 012106207 *Ovis aries*	...T.L....................L...G.......Q.V...............E.	[118]
	XM_ 012166124 *Ovis aries musimon*	...T.L....................L...G.......Q.V...............E.	[118]
	XM_ 010800305 *Bos taurus*	...T.L....................L...G.........V...............E.	[118]
	XM_ 005228505 *Bos taurus*	...T.L....................L...G.........V...............E.	[118]
	XM_ 012107055 *Ovis aries*	...T.L...........HY...........G.......Q.V...............EK	[118]
	XM_ 005895060 *Bos mutus*	...T.V...........HY...........G.......Q.V...............EK	[118]
	XM_ 010799571 *Bos taurus*	...T.L...........HY...........G.......Q.V...............EK	[118]
	XM_ 010821607 *Bos taurus*	...T.L...........HY...........G.......Q.V...............EK	[118]
	XM_ 012172886 *Ovis aries musimon*	...T.L...........HY...........G.......Q.V...............EK	[118]
	XM_ 013976867 *Capra hircus*	...T.L...........HY...........G.......Q.V...............EK	[118]
Aquatic mammals	XM_ 004318593 *Tursiops truncatus*	...T..............E.....................V..............S..	[118]
	XM_ 007172595 *Balaenoptera acutorostrata scammoni*	....V...........E..S..	[118]
