# Supplementary material for: Ruminant-specific multiple duplication events of PRDM9 before speciation
Source: BMC Evol Biol. 2017 Mar 14;17:79. doi: 10.1186/s12862-017-0892-4 (PMC5351255; doi:10.1186/s12862-017-0892-4)

Additional file 3. The PR domains of human PRDM7 and PRDM9 are aligned with the corresponding sequences of each lineage. The human PRDM7 (accession: Q9NQW5) and PRDM9 (accession: Q9NQV7) were recently reported (residues 244-358) in Blazer *et al.*, 2016. The three divergent residues in the PR domain between the human PRDM7 and PRDM9 (Blazer *et al.*, 2016) are highlighted in blue color. Amino acid residues unique to the PR domain of each lineage are colored coded.

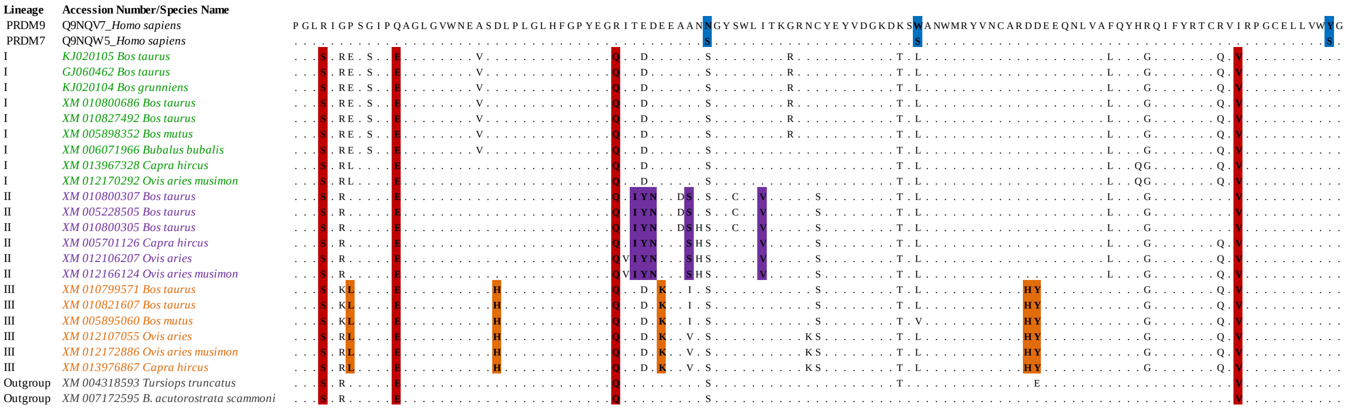

Supplement: Additional file 3: — The PR domains of human PRDM7 and PRDM9 are aligned with the corresponding sequences of each lineage. (PDF 96 kb) [file 12862_2017_892_MOESM3_ESM.pdf]
