# Supplementary material for: Ruminant-specific multiple duplication events of PRDM9 before speciation
Source: BMC Evol Biol. 2017 Mar 14;17:79. doi: 10.1186/s12862-017-0892-4 (PMC5351255; doi:10.1186/s12862-017-0892-4)
Supplement: Additional file 4: — GenBank accession numbers of the PRDM9 nucleotide sequences analyzed in this study. (PDF 80 kb) [file 12862_2017_892_MOESM4_ESM.pdf]

Additional file 4. GenBank accession numbers of the PRDM9 nucleotide sequences analyzed in this study.

KJ020105, GJ060462, KJ020104, XM_010800686, XM_010827492, XM_005898352, XM_006071966, XM_013967328, XM_012170292, XM_010800307, XM_005701126, XM_012106207, XM_012166124, XM_010800305, XM_005228505, XM_012107055, XM_005895060, XM_010799571, XM_010821607, XM_012172886, XM_013976867, XM_004318593, XM_007172595
